# Neurological Manifestations of Wilson's Disease: Pathophysiology and Localization of Each Component

**DOI:** 10.7759/cureus.11509

**Published:** 2020-11-16

**Authors:** Juan Fernando Ortiz, Álvaro Morillo Cox, Willians Tambo, Noha Eskander, Martín Wirth, Margarita Valdez, Maria Niño

**Affiliations:** 1 Neurology, California Institute of Behavioral Neurosciences & Psychology, Fairfield, USA; 2 Medicine, Universidad San Francisco de Quito, Quito, ECU; 3 Neurology, Universidad San Francisco de Quito, Quito, ECU; 4 Psychiatry, California Institute of Behavioral Neurosciences & Psychology, Fairfield, USA; 5 Internal Medicine, Universidad Autónoma de Guadalajara, Laredo, USA; 6 Emergency Medicine, Universidad del Rosario, Bogotá, COL

**Keywords:** wilson disease, dysarthria, tremor dystonia, tremor, parkinsonism, choreoathetosis, cerebellar ataxia, behavioral and psychiatric symptom, cognitive impairment

## Abstract

Wilson’s disease (WD) is an autosomal recessive disease that presents mainly with hepatic, neurological, and psychiatric manifestations. Neurological manifestations have been described in the past. Nevertheless, the pathophysiology and the clinical relevance of these manifestations have not been described in great detail in the medical literature. We aim to consolidate the knowledge about the neurological manifestations of WD and present the pathophysiology of each neurological manifestation of the disease. We will give a brief definition, the provenance, and the pathophysiology of the neurological conditions. We collected data from the National Library of Medicine (PubMed) using regular keywords and medical subject headings. Studies were selected applying the following inclusion/exclusion criteria: (1) studies that used exclusively human subjects, (2) papers published in English, and (3) papers from 1990 onward. The exclusion criteria were (1) studies that used animals, (2) papers not published in English, and (3) papers published before 1990. Additional studies were included via reference lists of identified papers and related articles featured in PubMed and Google Scholar.

Copper toxicity is the principal factor for brain degeneration seen in WD. Parkinsonism seen in WD has been associated with a nigrostriatal dopaminergic deficit. Resting tremor may have the same pathophysiology as parkinsonism. Action tremor is related to an accumulation of copper in the cerebellum's vermis and hemispheres. At the same time, essential tremor can be explained due to affection of the dentate nucleus. Choreoathetosis is produced due to increased activity of the direct pathway. We did not find specifically associated pathophysiology related to dysarthria. We assume that multiple parts of the brain are involved in that problem. Putamen nucleus damage is the leading cause that explains dystonia seen in WD along with the globus palidus. We did not find a specific localization for seizures in WD, but the pathology seems to be related to decreased levels of B6 and direct toxicity of copper on the brain.

## Introduction and background

Wilson’s disease (WD) is an autosomal recessive disorder due to a mutation of the chromosome on the ATP7B gene [1], which codes for a copper-transporting ATPase [1]. The transporter mediates the excretion of copper into bile and is essential to deliver copper for the synthesis of ceruloplasmin. This transporter's absence leads to the accumulation of copper in the liver, brain, and other organs [2]. The prevalence of WD is 1.7 patients per 100.000 [3]. The primary manifestations of WD are hepatic, neurologic, and psychiatric [3].

The main regions where copper is deposited in the brain are the basal ganglia, thalamus, cerebellum, and upper brainstem [2]. Neurological manifestations are expected in WD because of copper deposition on multiple regions of the brain. WD's main neurologic manifestations are dysarthria, dystonia, parkinsonism, tremor, choreoathetosis, ataxia, and cognition difficulties [3,4]. While the neurological manifestations of WD have been described, every individual component's pathophysiology has not been described fully in the literature. We aim to consolidate the knowledge about the neurological manifestations of WD and present the pathophysiology of each neurological manifestation of the disease. We will give a brief definition, the provenance, and the pathophysiology of the neurological manifestations.

## Review

Methods

Data is collected from the National Library of Medicine (PubMed) by means of regular keywords and medical subject headings (MeSH) strategy and other cross-references. The following MeSH terms were used with the subheading “Hepatolenticular degeneration” AND “Dystonia,” “Hepatolenticular degeneration” AND “Parkinsonism,” “Hepatolenticular degeneration” AND “dysarthria,” “Hepatolenticular degeneration” AND “Tremor.” “Hepatolenticular degeneration” AND “Choreoatetosis,” “Hepatolenticular degeneration” AND “Cognitive Impairment,” “Hepatolenticular degeneration” AND “Cerebellar Ataxia,” “Hepatolenticular degeneration” AND “Seizures.”

Table 1 shows the total records and selected records for the review of the article.

**Table 1:** Total initial and final selected records for discussion of the paper.

MeSH Terms	Total Records	Records Selected
“Hepatolenticular degeneration” AND “Dystonia”	43	4
“Hepatolenticular degeneration” AND “Parkinsonism”	46	7
“Hepatolenticular degeneration” AND “Dysarthria”	20	1
“Hepatolenticular degeneration” AND “Tremor”	41	3
“Hepatolenticular degeneration” AND “Choreoathetosis”	2	1
“Hepatolenticular degeneration” AND “Cognitive Impairment”	2	1
“Hepatolenticular degeneration” AND “Cerebellar Ataxia”	16	2
“Hepatolenticular degeneration” AND “Seizures”	23	2

Studies were selected by applying the following inclusion/exclusion criteria: (1) studies that used exclusively human subjects, (2) papers published in English, and (3) papers published after 1990. The exclusion criteria were (1) studies that used animals, (2) papers not published in English, and (3) papers published before 1990. Additional studies were included via reference lists of identified papers and related articles featured in PubMed and Google Scholar.

Results

Initially, we identified 277 articles; but after applying the inclusion/exclusion criteria, we ended up with 28 articles. Table 2 outlines the MeSH search strategy and resulting articles.

**Table 2:** Summary of the search results of this study.

Search Results	193
English language	176
Human subjects	175
Free full text	68
Exclusion of papers of more than 30 years	28

After selecting 28 articles, we excluded another nine articles due to one of the following reasons:

1. Studies did not have the desired outcome.

2. Extraction data of the article was not possible.

3. There was a duplication of data.

Finally, we used 19 articles for discussion, and nine additional studies were added via reference lists of identified papers and related articles featured in PubMed and Google Scholar, thus making the discussion to a total of 28 articles.

Discussion

In the brain of WD's patients, the most common macroscopic abnormalities are found in the basal ganglia, specifically in the dorsal striatum, but also has been described in many other sites such as the thalamus, brainstem, and frontal lobe as well [5]. The most common magnetic resonance imaging (MRI) findings were areas of high T2 signal in the lentiform and caudate nuclei, thalamus, brainstem, and white matter [6].

For this review, we discuss the main neurological manifestations of WD: dysarthria, dystonia, parkinsonism, tremor, cognitive impairment, and choreoathetosis. The prevalence of the neurologic manifestations is a study by Machado et al. in decreasing order as follows: dysarthria 91%, dystonia 69%, Parkinson's symptoms (rigidity 66%, resting tremor 5%, bradykinesia 58%), cerebellar disturbances 28%, chorea 16%, and athetosis 14%. The prevalence of cognitive impairment is rare with a frequency of 4.2% [4].

Parkinsonism

Parkinsonism is a clinical syndrome, which manifests with bradykinesia, postural instability, rest tremor, and rigidity. The most common cause of this syndrome is idiopathic Parkinson's disease (PD). Nevertheless, other etiologies must be considered, such as Parkinson's plus syndromes (multiple system atrophy, corticobasal degeneration, and progressive supranuclear palsy) and secondary parkinsonism (neuroleptics side effects, traumatic brain injury, and metoclopramide) [7,8]. In WD, parkinsonism includes bradykinesia, imbalance, and cogwheel rigidity and usually presents symmetric fashion. This deferred from the atypical asymmetrical presentation of PD [2].

Parkinsonism in WD is produced due to the nigrostriatal dopaminergic deficits. Cerebellar symptoms and dysphagia help to differentiate PD from parkinsonism in WD [6]. Interestingly, patients with WD and parkinsonism do not respond to levodopa [6]. Levodopa acts mainly in presynaptic receptors. In WD, there is damage in the presynaptic and the postsynaptic dopamine receptors. This alteration could explain the lack of response to levodopa in WD [6,9].

A critical correlation between PD and WD is that there are low levels of ceruloplasmin in PD. Reduced ceruloplasmin levels lead to copper and iron accumulation. Additionally, liver dysfunction may also lead to the accumulation of manganese. The collection of these metals in the brain causes neurodegeneration. Degeneration of subcortical structures leads to parkinsonism and other neurological symptoms [10].

It has been found in patients with WD hyperintensities in the basal ganglia, and it reduces glucose uptake in the striatum. Patients treated with penicillamine showed decreased hyperintensities on T2-weighted imaging on basal ganglia. Glucose reuptake levels came back to normal in these patients as well [11]. Another report found a correlation between parkinsonism in WD and hyperintense lesions on T2 and low intensity on T1 MRI [11].

Ataxia

Ataxia describes signs and symptoms resulting from cerebellar dysfunction and the affectation of cerebellar pathways. Ataxia involves abnormalities of the posture, gait, dysdiadochokinesia, dysmetria, hypotonia, oculomotor abnormalities, tremor, and speech disturbances [12].

In WD, copper levels are increased nonselectively in all brain regions. The compromise of the dentate nucleus of the cerebellum can explain the presence of ataxia in these patients. At the same time, some authors have reported relationships between tremor and ataxia with globus pallidus lesions. It is essential to consider that profound demyelination has been described in copper toxicity. The demyelination suggests that the cerebellar tracts' damage contribute to the pathophysiology [13,14].

Other studies have correlated MRI lesions in the brainstem, cerebellum, and cerebral cortex with ataxia. Cerebellar dentate nucleus T2 hypointensity on MRI is a characteristic finding as well as cerebellar atrophy. Focal thalamic lesions have also been associated with this affection. Besides, MRI can demonstrate unspecific white-matter changes [15].

It is important to remember that balance and coordination are a complex circuitry product involving the basal ganglia, cerebellum, cerebral cortex, peripheral motor, and sensory pathways. The dysfunction of any part of this integrated system can lead to ataxia [16]. The dysfunction correlates with the lesions described before and explains why this manifestation is seen in WD.

Tremor

Tremor is defined as an involuntary, rhythmic, oscillatory movement of a body part. In hepatolenticular degeneration and other neurological diseases, pathological tremor is usually persistent and visible. Most of them have a frequency of 4-8 Hz with variable amplitude [17]. In some series, this clinical feature is reported as the most frequent neurological manifestation of WD, including postural, rubral (wing-beating), and rest tremors. It usually starts in one limb and may eventually spread to the whole body. Proximal tremor of high amplitude or wing-beating tremor is a characteristic symptom of WD. Postural tremor occurs when a specific position or posture is voluntarily maintained, while the rest tremor is the opposite [3,4].

We believe that resting tremor, which is primarily seen in parkinsonism, has the same physiopathology as described above in PD. The action tremor in WD occurs due to copper deposition principally in the cerebellum. It frequently accumulates in the vermis and hemispheres, producing ataxic gait, dysdiadochokinesis, and compromised fine hand movement. The tremor starts in the distal upper extremities unilaterally. As the disease progresses, the head, the legs, and the whole body are affected [18]; this is consistent with how the distal muscles are coordinated with the intermediate zone of the cerebellar hemispheres adjacent to the vermis. Copper accumulates in larger quantities affecting the vermis's cortex; this compromises the coordination and movements of the neck, shoulders, thorax, abdomen, and hips. On neuroimaging, there are usually hypointensities in these mentioned structures, and it can be accompanied by cerebellar atrophy in later stages of the disease [10].

Another common type of tremor seen in WD is an essential tremor. The literature reports that essential tremor exists due to neurodegeneration in the cerebellar dentate nucleus. This neurodegeneration produces a gamma-aminobutyric acid (GABA) dysfunction in the cerebellothalamocortical circuit [19]. This fact is coherent with hypointensities reported in MRI neuroimaging in the cerebellar dentate nucleus and brain stem. One study found associations between tremor and cerebellar atrophy and ataxia with lesions in the brain stem, cerebellum, and cerebral cortex [20]. The study also found that patients between 11 and 20 years of age at the beginning of the disease were at higher risk of forming thalamic lesions. In contrast, patients between 21 and 31 years of age were more expected to have cerebellar lesions [20].

Dystonia

Dystonia describes a neurological condition characterized by involuntary sustained or intermittent muscle contractions producing repetitive movements and abnormal postures. It can be the manifesting neurological sign of many disorders, including WD [21].

The clinical manifestation of dystonia in WD is broad. It ranges from mild cases to severe disease and could manifest as focal, segmental, multifocal, or generalized symptoms. Focal manifestation includes torticollis, blepharospasm, and risus sardonicus [2]. The last manifestation is the most characteristic presentation and manifests as a fixed smile due to the dysfunction of the risorius muscle [2]. Likewise, focal dystonia of the vocal cords and articulation muscle may produce dysphonia, dysarthria, and dysphagia. At the early stage of the disease, the predominant location of symptoms is unilateral. As the disease progresses, it turns bilateral and often generalized [3].

Interestingly, dystonia correlates with imaging abnormalities viewed on MRI signal in the putamen [3]. The putamen is the main structure involved in the dystonic movements suggesting a strong correlation between the clinic and this brain region [22]. Damage in the basal ganglia-thalamocortical motor circuits fails to generate an inhibitory signal in the cortical neurons producing an excessive motor output and increasing activity of the direct pathway of the basal ganglia [22,23]. Finally, it is essential to mention that dystonia is not only a basal ganglia disease. Dystonia also has a cerebellar and cortical disease component [23].

Choreoathetosis

Chorea is characterized by dance-like movements with rapid and unpredictable contractions, usually in distal limbs, but it can also compromise proximal limbs, face, and trunk. On the other hand, athetosis refers to slow writhing movements involving distal extremities and other body parts, such as the face. The term choreoathetosis is used when typical choreic movements coexist with athetosis. It seems that the basal ganglia are the main structures involved in its pathophysiology [24,25].

High levels of copper disrupt the hematoencephalic barrier and deposit at the striatum, globus pallidus, locus coeruleus, substantia nigra, and cerebral cortex [18,26]. Accumulation of copper in this structure caused choreoathetosis. Copper induces oxidative damage from free radicals and lipid peroxidation, consequently developing necrosis, extensive gliosis, and detrimental neuronal loss [13,26].

All the changes mentioned earlier cause suppression in the function of the globus pallidus over the thalamus-cortex-brain stem pathway. The direct pathway gets affected, causing overstimulation leading to hyperkinetic choreoathetosis movements [13,27]. Such disruptive functional alterations are also evident in MRI. MRI is a diagnostic and prognostic tool that identifies structural changes, notably at basal ganglia, thalami, and brainstem. There is no consensus within the different scales, but we can use other imaging findings to classify disease progression. Certain assumptions with correlation to a clinical stage can be made. Mild T2 hyperintensities represent changes that may be reversible with treatment. In contrast, the following imaging represents irreversible changes: T1 hypointensities, atrophy, and T2 hypointensities (suggesting iron depositions). These changes have a worse prognosis [18].

Cognitive impairment

Cognitive changes have been reported in WD, including frontal syndrome and subcortical dementia. The frontal syndrome results from frontal lobe degeneration, which presents a disorder of the executive function and behavior changes [28]. Simultaneously, subcortical dementia is characterized by memory loss, personality changes, mood disturbances, and slowness of mentation [29]. It is rare to find a patient with defects in just one aspect. Instead, it may present as a combination of frontal lobe syndrome and subcortical dementia [4,13].

The brain's main pathological changes were caused by copper deposition. The main changes we find were hypertrophy of the astrocytes, cerebral edema, cystic changes, and demyelination. Regarding imaging, lesions tend to be symmetrical and bilateral [20]. Lesions in the cortex are more common in the frontal lobe, which would explain the frontal lobe syndrome. It is important to note that white and gray matters were affected. Lesions in the basal ganglia, midbrain, and pons could suggest evidence of subcortical dementia [20].

Dysarthria

Dysarthria is a motor speech disorder characterized by imprecise, slow, weak, and uncoordinated speech movements. It can result from any condition that damages the motor control structures necessary for speech production, including lower motor neuron lesions of cranial nerves IX, X, and XII, cerebellar or basal ganglia disorders. All speech mechanism components may be affected in different manners, such as features associated with articulation, phonation, prosody, respiration, and resonance [30]. As said before, dysarthria is caused by multiple regions of the brain. We did not find a specific pathophysiologic mechanism for dysarthria.

Seizures

A study of 110 patients with WD and neurological manifestations found that 16 patients (14.5%) had seizures; 68.7% of those patients had partial seizures, and the other 31.3% generalized seizures [31]. The electroencephalogram (EEG) found epileptiform discharges in 10 out of 16 patients. Seven patients required one anti-epileptic medication (AED) to control the seizures; seven patients required two AED; and two patients required three medications for seizure control. Another report found 4% of patients having seizures after treatment (penicillamine) [32]. The mean levels of copper levels were higher in patients with seizures (35.87) vs. patients without seizures (31.72) [31]. MRI cortical, subcortical, and cerebellar lesions were found more commonly in patients with seizures than in patients without them. There are four theories regarding the pathophysiology of seizures in WD. First, penicillamine treatment can cause pyridoxine deficiency. Decreasing levels of pyridoxine decreased the levels of GABA; decreased levels of GABA lower the seizure threshold. Seizures in WD can also occur due to the direct toxicity of copper due to inhibition of membrane ATPase [32]. The presence of copper generates oxidative stress, which releases glutamate and pro-inflammatory cytokines [31]. Due to the significant number of lesions found in patients with WD seizures, we cannot specify a particular region responsible for the seizures. It is important to note that MRI findings with lesions in the frontal lobe were the most common findings in patients with seizures and WD [31].

## Conclusions

It is well known that copper toxicity is the principal reason responsible for brain degeneration seen in WD. As reported, this metal can be found in every encephalon region and cause different manifestations described above. Parkinsonism seen in WD has been associated with the nigrostriatal dopaminergic deficit and reduced ceruloplasmin levels, leading to iron and copper accumulation. Imaging abnormalities associated with basal ganglia have been described. Resting tremor may have the same pathophysiology as parkinsonism. In comparison, action tremor is related to an accumulation of copper in the cerebellum's vermis and hemispheres. Essential tremor is caused due to affection of the dentate nucleus. Ataxia responds to lesions in the brainstem, basal ganglia, cerebellum (dentate nucleus), cerebral cortex, and white matter. Putamen nucleus damage is the leading cause that explains dystonia seen in WD along with globus pallidus. Copper deposits in the striatum, globus pallidus, locus ceruleus, substantia nigra, and cerebral cortex justify the presence of choreoathetosis. Lesions in the brain cortex, mostly frontal, and white matter as well as other profound structures correlate with cognitive impairment. We did not find specific pathophysiology related to dysarthria in WD. Different parts of the brain must be involved in the pathophysiology of dysarthria. We did not find a specific localization for seizures in WD, but the pathology seems to be related to decreased levels of B6 and direct toxicity of cooper on the brain. This article describes the evidence that can explain WD's neurological manifestations, but further research is needed to fill gaps in knowledge that are still present.
